# Detection and isolation of a new member of *Burkholderiaceae*-related endofungal bacteria from *Saksenaea boninensis* sp. nov., a new thermotolerant fungus in *Mucorales*

**DOI:** 10.1186/s43008-023-00129-2

**Published:** 2023-11-23

**Authors:** Yusuke Takashima, Kohei Yamamoto, Yousuke Degawa, Yong Guo, Tomoyasu Nishizawa, Hiroyuki Ohta, Kazuhiko Narisawa

**Affiliations:** 1 Genetic Resources Center, National Agriculture and Food Research Organization, 2-1-2 Kannondai, Tsukuba, Ibaraki 305-8602 Japan; 2 Mountain Science Center, Sugadaira Research Station, University of Tsukuba, Sugadaira-kogen 1278-294, Nagano, 386-2204 Japan; 3 Ibaraki University College of Agriculture, 3-21-1 Chuo, Ami-machi, Ibaraki 300-0393 Japan; 4 Tochigi Prefectural Museum, 2-2 Mutsumi-cho, Utsunomiya, Tochigi 320-0865 Japan; 5 Ibaraki University, 2-1-1 Bunkyo, Mito, Ibaraki 310-8512 Japan; 6 Present Address: Institute for Plant Protection, National Agriculture and Food Research Organization, 2-1 Fujimoto, Tsukuba, Ibaraki 305-8605 Japan

**Keywords:** Endohyphal bacteria, Mucormycosis, *Mucoromycotina*, One new taxon, Opportunistic pathogens, *Saksenaeaceae*, Thermotolerant fungi

## Abstract

Thermotolerance in *Mucorales* (*Mucoromycotina*) is one of the factors to be opportunistic pathogens, causing mucormycosis. Among thermotolerant mucoralean fungi, *Burkholderiaceae*-related endobacteria (BRE) are rarely found and the known range of hosts is limited to *Rhizopus* spp. The phylogenetic divergence of BRE has recently expanded in other fungal groups such as *Mortierellaceae* spp. (*Mortierellomycotina*); however, it remains unexplored in *Mucorales*. Here, we found a thermotolerant mucoralean fungus obtained from a litter sample collected from Haha-jima Island in the Ogasawara (Bonin) Islands, Japan. The fungus was morphologically, phylogenetically, and physiologically characterized and proposed as a new species, *Saksenaea boninensis* sp. nov. Besides the fungal taxonomy, we also found the presence of BRE in isolates of this species by diagnostic PCR amplification of the 16S rRNA gene from mycelia, fluorescence microscopic observations, and isolation of the bacterium in pure culture. Phylogenetic analysis of the 16S rRNA gene of BRE revealed that it is distinct from all known BRE. The discovery of a culturable BRE lineage in the genus *Saksenaea* will add new insight into the evolutional origin of mucoralean fungus-BRE associations and emphasize the need to pay more attention to endofungal bacteria potentially associated with isolates of thermotolerant mucoralean fungi causing mucormycosis.

**Supplementary Information:**

The online version contains supplementary material available at 10.1186/s43008-023-00129-2.

## Introduction

Bacterial endosymbionts are commonly found in eukaryotes including fungi, and such fungus-bacterium interactions have been widely recognized in mycology and environmental microbiology (Bonfante and Desirò 2017; Robinson et al. 2021). These bacterial endosymbionts are known as endofungal or endohyphal bacteria, in which the family *Burkholderiaceae* associated with *Mucoromycota* is the most extensively studied bacterial lineage (Bonfante and Desirò 2017). Each bacterial genus of *Burkholderiaceae*-related endobacteria (BRE) such as *Mycetohabitans* spp., *Mycoavidus* spp., and ‘*Candidatus* Glomeribacter gigasporarum’ was found in three subphyla: *Mucoromycotina*, *Mortierellomycotina*, and *Glomeromycotina*, respectively (Bonfante and Desirò 2017). BRE associated with *Mortierellaceae* (*Mortierellomycotina*) and *Gigasporaceae* (*Glomeromycotina*) fungi are phylogenetically diverse, and the host ranges of each BRE lineage cover multiple genera and species (Desirò et al. 2014; Mondo et al. 2012; Takashima et al. 2018). Among *Mucorales*, the most known fungus that harbors endohyphal bacteria (namely *Mycetohabitans* spp.) is *Rhizopus microsporus* (Partida-Martinez et al. 2007). *Rhizopus microsporus*, because of its thermotolerant characteristic, is one of the opportunistic pathogens of humans and other animals, as it can cause mucormycosis. The prevalence of BRE in clinical strains and isolates, as well as other thermotolerant mucoralean fungi remains however understudied.

One of other thermotolerant *Mucorales* is *Saksenaea* spp., which are occasionally found in soil, water, and clinical specimens of animals and humans (Ajello et al. 1976; Alvarez et al. 2010; Crous et al. 2016; Crous et al. 2017; Nam et al. 2021; Saksena 1953; Singh and Kushwaha 2017). Similar to *Rhizopus* spp., some species of *Saksenaea* are capable of growing rapidly at human body temperature and even at over 40 °C (Baijal 1967; Alvarez et al. 2010; Crous et al. 2017; Labuda et al. 2019). This is one of the traits allows *Saksenaea* species to become opportunistic causal agents of mucormycosis (Ajello et al. 1976; Chakrabarti and Singh 2014; Chander et al. 2018; Roden et al. 2005)—a growing problem, especially in the midst of the pandemic of COVID-19 (Hoenigl et al. 2022).

In the present study, four isolates of *Saksenaea* were obtained from a litter sample collected from Haha-jima Island in the Ogasawara (Bonin) Islands, with uniquely endemic fauna and flora that originated from isolated locations far from the main islands of Japan as oceanic islands. These isolates were morphologically, phylogenetically, and physiologically investigated. These analyses resulted in describing a new species from the genus *Saksenaea*: we propose *Saksenaea boninensis* sp. nov. We also detected intracellular bacteria in this species by fluorescence microscopy using nucleic staining and fluorescence in situ hybridization (FISH), and isolation of the bacteria in pure cultures. Phylogenetic analysis showed that the detected bacteria formed a single lineage within the family *Burkholderiaceae*, but were distinct from all known BRE.

## Materials and methods

### Fungal isolation

**Fig. 1:**
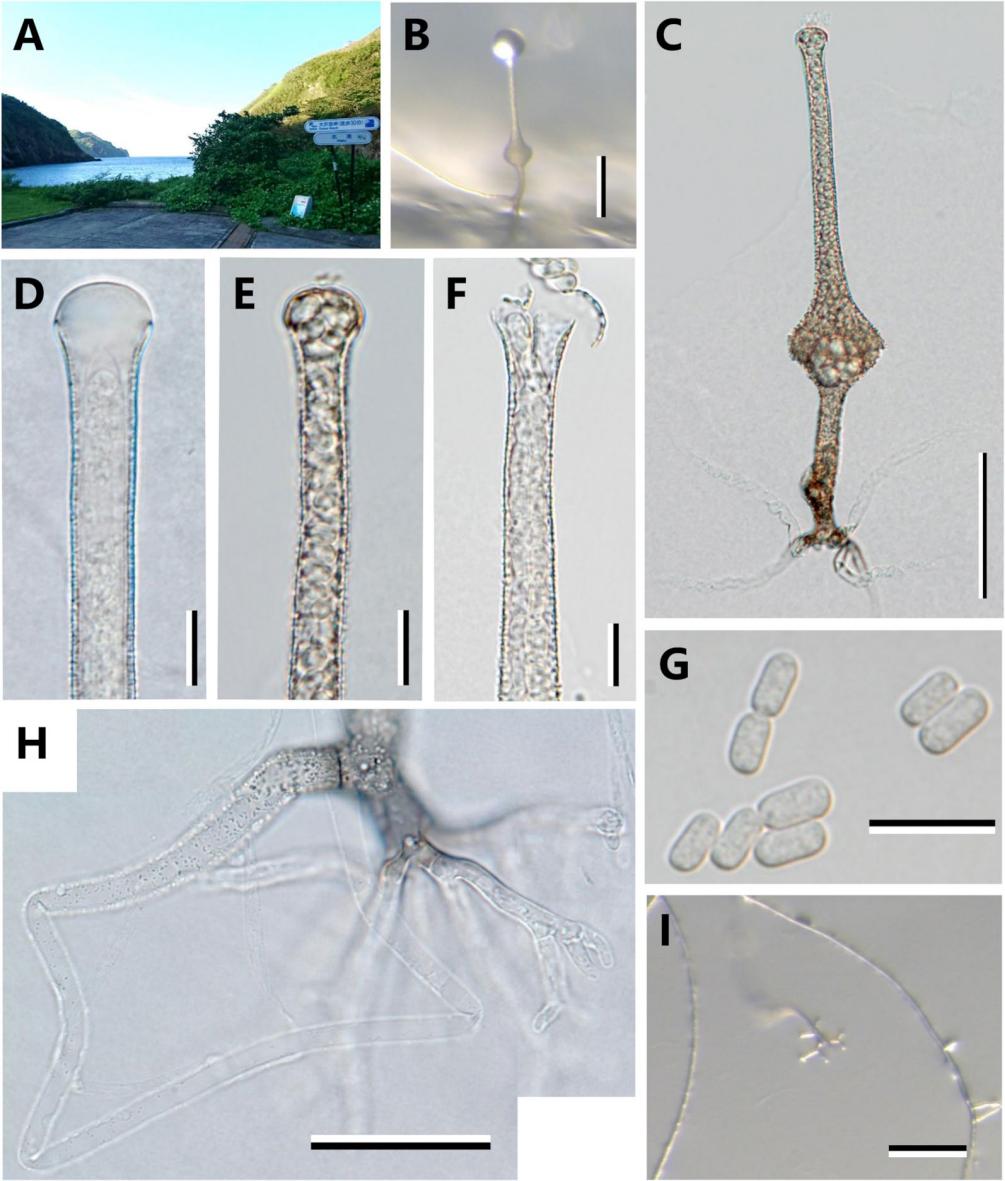
The landscape of collection site and microscopic characters of *Saksenaea boninensis* Sak4. The landscape of Haha-jima Island where the litter was collected **A** The morphologies of the isolate were observed after incubation for 1 month on CZA at 23 °C, unless otherwise specified. **B**–**I**. **B**: A sporangium developed from a stolon. **C** A sporangium. **D**–**F**: Stages of sporangia showing formation of the apical portion of the neck with a mucilaginous plug after incubation for 7 d **D**, the gradual dissolution of apical mucilage **E**, and liberation of sporangiospores **F**. **G** Sporangiospores. **H**: A dichotomously branched rhizoid partitioned by a septum from a stolon. **I**: An early stage of a rhizoid formed when the tip of the stolon touched the surface of the medium. Scale bars: **B**, **I** 100 μm; **C** 50 μm; **D**–**G** 10 μm; **H** 30 μm

Litter was collected from the Kitako port (north port) of Haha-jima Island in the Ogasawara (Bonin) Islands, Tokyo, Japan (N 26° 41′ 40.4", E 142° 08′ 49.7") on 9th November, 2018 (Fig. 1A). The litter was directly spread onto _LC_A medium (Miura medium) [0.2 g yeast extract (Difco, Sparks, MD, USA), 1 g glucose (Wako Pure Chemical Industries, Osaka, Japan), 2 g NaNO_3_ (Wako), 1 g KH_2_PO_4_ (Wako), 0.2 g KCl (Wako), 0.2 g MgSO_4_·7H_2_O (Wako), 15 g Bacto agar (Difco) in 1 L distilled water] (Miura and Kudo 1970) and incubated at room temperature (*ca.* 23 °C) under ambient light conditions in the laboratory. After 14-d incubation, sporangia of *Saksenaea* were produced throughout the surface of a single agar medium. Consequently, it was not known whether each sporangium was identical as an individual. Four isolates were established by inoculating sporangiospores in each randomly selected sporangium onto fresh _LC_A medium using a frame-sterilized fine needle and incubated at 30 °C before use for further analyses. The consistent sporulation of each isolate was found in Czapek-Dox Agar medium (CZA) [30 g sucrose (Wako), 2 g NaNO_3_ (Wako), 1 g K_2_HPO_4_ (Wako), 0.5 g MgSO_4_·7H_2_O (Wako), 0.5 g KCl (Wako), 0.01 g FeSO_4_·7H_2_O (Wako), 15 g Bacto agar (Difco) in 1 L distilled water]; however, the isolate Sak4 was practically selected as the well-sporulated representative isolate in a tentative sporulation observation using a sporulation method by Padhye and Ajello (1988). For the preparation of a dried specimen, the representative isolate Sak4 was incubated on CZA for 1 month for sporulation and then inactivated by complete drying using a drying oven at 60 °C for 3 d, and deposited in the Kanagawa Prefectural Museum of Natural History (KPM, Kanagawa Pref., Japan). A living culture of the isolate was deposited in the NARO Genebank (MAFF, Ibaraki Pref., Japan), the Japan Collection of Microorganisms (JCM, Ibaraki Pref., Japan), the NITE Biological Resource Center (NBRC, Chiba Pref., Japan), and the CBS-KNAW culture collection (CBS, Utrecht, The Netherlands).

### Colony morphology and growth measurement

Prior to the measurement, the representative isolate Sak4 was pre-incubated on _LC_A medium for 4 or 10 d at 30 °C. Then, a disc was cut out from the incubated mycelia using an autoclave-sterilized plastic straw (8 mm diam) as a substitute for a cork borer and placed onto CZA (90-mm-diam Petri dish) with at least three replicates. The plates were incubated for 4 d at 13, 15, 18, 23, 25, 28, 30, 35, 37, 38, 40, and 42 °C and the colony diameter was measured daily. For the comparisons of culture characteristics, a disc of the representative isolate Sak4 pre-incubated on CZA medium for 4 d at 30 °C was cut out as the same way above and placed onto Corn Meal Agar medium (CMA; Nissui Pharmaceutical Co., Ltd., Tokyo, Japan), CZA, _LC_A, Malt Extract Agar medium (MEA; Difco), and Potato Dextrose Agar medium (PDA; Nissui). Colony characteristics on each medium were observed after the incubation at the optimum growth temperature (30 °C) for 3 d.

### Morphological observation

The representative isolate Sak4 was incubated for 1 month on CZA for sporulation at 23 °C. Morphological observation was carried out using a stereomicroscope (M205C, Leica, Germany) and a light microscope (BX51, Olympus Corp., Tokyo, Japan) equipped with a digital camera (DP25, Olympus Corp.). The fungal materials were mounted in distilled water or lactic acid.

### DNA extraction, PCR, and sequencing of fungal isolates

Template DNA was extracted from seven-day-old mycelia of each isolate incubated on a sterilized cellophane sheet placed in half-strength Corn Meal-Malt-Yeast Agar medium (1/2 CMMY) [8.5 g corn meal agar (Difco), 10 g malt extract (Difco), 1 g yeast extract (Difco), 7.5 g Bacto agar (Difco) in 1 L distilled water] using the Prepman™ Ultra sample reagent (Applied Biosystems, Foster City, CA, USA) in accordance with Sato et al. (2010). The ITS1-5.8S-ITS2 and partial large subunit ribosomal RNA (LSU) gene regions and the partial transcription elongation factor 1-α (*tef1*) gene of each isolate were also amplified. Regarding the PCR amplification of ITS1-5.8S-ITS2 and partial LSU regions, 50 μL of a PCR mixture containing 1.0 μL of template DNA, 1.5 μL of each primer solution of the fungal universal primers ITS5 and LR5 (10 pmol μL^−1^ each, White et al. 1990; Vilgalys and Hester 1990), 10 μL of 2 mM dNTPs, 1.0 μL of 1.0 U μL^−1^ KOD FX Neo DNA polymerase (Toyobo, Osaka, Japan), 25 μL of 2 × PCR Buffer for KOD FX Neo DNA polymerase, and 10 μL of sterilized deionized water was prepared. PCR amplification was performed as follows: initially 2 min for 94 °C, followed by 30 cycles of 98 °C for 10 s, 58 °C for 30 s, and 68 °C for 1 min using a thermal cycler. Cycle sequence reaction was performed with a BigDye Terminator Cycle Sequencing Ready Reaction Kit (Applied Biosystems) following the manufacturer’s instructions. Cycle sequencing of the PCR products of ITS5-LR5 was performed using ITS5, ITS3, ITS4, LR0R, and LR5 primers (White et al. 1990; Vilgalys and Hester 1990). For the partial *tef1* gene, the PCR mixtures as described above with two primer sets, 526F-1567R and 983F-2218R (Rehner and Buckley 2005), were separately prepared. PCR amplification conditions were the same as those shown above except the annealing temperatures were set as 55 °C and 58 °C, respectively, and the number of cycles was set as 35. Cycle sequencing of the PCR products of 526F-1567R and 983F-2218R primer sets was performed using 526F and 1567R, and 983F, 2218R, and MEF11 primers, respectively (Rehner and Buckley 2005; O'Donnell et al. 2001). Cycle sequencing products were purified by ethanol precipitation, and electrophoresis was performed using the Applied Biosystems 3130xl genetic analyzer (Applied Biosystems) to determine nucleotide sequences. The DNA sequences obtained from each primer of each gene region (DNA sequences using 526F and 983F as sequencing primers were not obtained) were assembled into a single sequence using GeneStudio Professional software version 2.2.0.0 (http://www.genestudio.com/).

### Diagnostic PCR of the endofungal bacterium

DNA extracted from fungal mycelia was also used for detection of the 16S rRNA gene of the endofungal bacteria. For PCR amplification of the 16S rRNA gene, a PCR mixture with the bacterial universal primers 10F and 1541R (Takashima et al. 2018) was prepared and PCR amplification was performed under the same conditions as described in Takashima et al. (2018). Cycle sequencing of PCR products of 10F-1541R was performed using 10F, 341F, 800F, 926R, and 1541R primers (Lane et al. 1991; Muyzer et al. 1993; Takashima et al. 2018). DNA sequencing and assembly of the single sequence were performed in the same way as above.

### Fluorescence microscopy

To observe endofungal bacteria inside fungal cells, we performed fluorescence microscopic observations using the nucleic staining reagent LIVE/DEAD™ BacLight™ Bacterial Viability Kit (Molecular Probes, Eugene, OR, USA) and FISH with the Cy3-labeled bacterial universal probe EUB338 in accordance with Takashima et al. (2018). All fluorescence images were obtained using a fluorescence microscope (BX51, Olympus Corp., Tokyo, Japan) equipped with the digital camera EOS kiss X7i (Canon, Tokyo, Japan). Over exposure and contrast throughout each image were adjusted by Adobe Photoshop CS6 (Adobe Inc., San Jose, CA, USA).

### Isolation of the endofungal bacterium

Seven-day-old mycelia of each isolate incubated on a sterilized cellophane sheet placed in 1/2 CMMY agar at 30 °C were axenically collected from 3 plates (55-mm diam). Fresh mycelia (300–370 mg) of each isolate were homogenized by pestle and filtrated using jointed 8-µm and 3-µm membrane filters following the method of Desirò et al. (2023). One milliliter of the filtrated bacterial suspension of each isolate was separately added to buffered charcoal yeast extract with 0.1% α-ketoglutalate (BCYEα) medium (Oxoid, Hampshire, UK) and incubated at 23 and 30 °C. After 7-d incubation, a drop of the liquid layer of the culture plates incubated at 30 °C was streaked onto fresh BCYEα medium and then a single colony was obtained after 5-d incubation at 30 °C. Seven-day-old culture on BCYEα medium at 30 °C of the endofungal bacterium of each fungal isolate was used for DNA extraction with lysozyme, as in our previous study (Sharmin et al. 2018). Extracted DNA was used for PCR of the 16S rRNA gene, and the sequences were determined in the same way as above.

### Culturability of the endofungal bacterium

The bacterial isolate was pre-incubated on BCYEα medium at 30 °C for 7 d. Then, the colony on the medium was inoculated onto media including BCYEα medium, Luria–Bertani (LB) agar [25 g Luria–Bertani broth (Difco), 15 g agar (Difco) in 1 L distilled water], 1/10 nutrient broth agar [0.8 g nutrient broth (Difco), 15 g agar (Difco) in 1 L distilled water], R2A agar [3.2 g R2A Broth ''DAIGO'' (Nissui), 15 g agar (Difco) in 1 L distilled water]. These inoculated plates were incubated at 30 °C for 14 d and colony growth was checked after 5, 10, and 14 d.

### Phylogenetic analyses of the fungal host and endofungal bacterium

For the fungal host, ITS1-5.8S-ITS2, LSU, and *tef1* gene sequences of *Saksenaea* spp. and *Apophysomyces elegans* were obtained from GenBank (Table 1). Among 54 strains of *Saksenaea* spp. including four isolates obtained in this study, all three gene regions were present in 24 strains; however, the ITS2 region (177–189 bp) is only available in five strains of *S. longicolla*. Pairwise distances of the nucleotide sequences (ITS2, ITS1-5.8S-ITS2, LSU, and *tef1*) of the ex-type strains of seven *Saksenaea* spp. and the representative isolate Sak4 were calculated by MEGA 6.06 software (Tamura et al. 2013), and the percentages of the nucleotide divergence among species showed different patterns between the ITS2 only and the ITS1-5.8S-ITS2 region (Additional file 1: Table S1). Therefore, to compare tree topologies, we prepared two concatenated datasets containing ITS2, LSU, and *tef1* (dataset 1), or instead of ITS2 only, using ITS1-5.8S-ITS2 region by substituting those sequences of *S. longicolla* as blank sequences (dataset 2). The retrieved GenBank sequences and ITS2 (or ITS1-5.8S-ITS2), LSU, and *tef1* gene sequences obtained from the fungal isolates were aligned independently for each region using MAFFT v7.212 (Katoh and Standley 2013). The obtained alignment blocks were subject to Gblocks v0.91b (Castresana 2000) to remove poorly aligned positions with the relaxed selection setting described in Talavera and Castresana (2007) using the following parameters (−t = d −b2 = 9 −b3 = 10 −b4 = 5 −b5 = h). After automatically removing gaps, the alignment blocks were viewed using MEGA 6.06 software and poorly aligned positions at either end of the alignments were removed manually. The alignment blocks for each gene region were then concatenated [168 positions for ITS2 (or 554 positions for ITS1-5.8S-ITS2), 637 positions for LSU, and 471 positions for *tef1*, resulting in 1,276 positions in the dataset 1 and 1,662 positions in the dataset 2) and compared using the partition model in Kakusan4 (released on 4.0.2015.01.23; Tanabe 2011). All ML phylogenetic analyses were performed using RAxML version 8.1.5 (Stamatakis 2014) with a 1000 bootstrap replicates. For the concatenated datasets, the partition model was set as the “separate” model for dataset 1 and “equalrate” model for dataset 2 selected (lowest AIC) by Kakusan4. The nucleotide substitution model for the concatenated datasets was set as the GTRGAMMA model following the default setting of Kakusan4. For each region (ITS1-5.8S-ITS2, LSU, and *tef1*), the nucleotide substitution model was set as the GTRGAMMAI model selected in MEGA 6.06. *Apophysomyces elegans* was used as the outgroup in all phylogenetic analyses.

**Table 1:** Newly determined sequence of *Saksenaea boninensis* (bold) and sequences retrieved from GenBank for phylogenetic analyses

Taxon	Strain No.		ITS	LSU	*tef1*
*Apophysomyces elegans*	CBS 476.78, NRRL 22325	ex-type	FN556440	FN554249	AF157231
*Saksenaea boninensis*	Sak3		**MK757862**	**MK757858**	**LC474956**
*Saksenaea boninensis*	Sak4, MAFF 247844, JCM 39173, NBRC 114970, CBS 147591	ex-type	**MK757863**	**MK757859**	**LC474957**
*Saksenaea boninensis*	Sak5		**MK757864**	**MK757860**	**LC474958**
*Saksenaea boninensis*	Sak6		**MK757865**	**MK757861**	**LC474959**
*Saksenaea dorisiae*	BiMM-F232	ex-type	MK559697	MK570305	MK569515
*Saksenaea erythrospora*	CBS 138279		KM102733	KM102734	KM102735
*Saksenaea erythrospora*	CIMCE001		KU951560	–	–
*Saksenaea erythrospora*	FMR 13392		KR527481	–	–
*Saksenaea erythrospora*	FMR 13516		KR527482	–	–
*Saksenaea erythrospora*	FMR 13880		KR527483	–	–
*Saksenaea erythrospora*	M-1024/14		–	KR527484	–
*Saksenaea erythrospora*	M-340/14		–	KR527485	–
*Saksenaea erythrospora*	RTCC239110910		JF433911	JF433912	-
*Saksenaea erythrospora*	UTHSC 06-576		FR687331	HM776683	HM776694
*Saksenaea erythrospora*	UTHSC 08-3606	ex-type	NR_149333	NG_059935	HM776691
*Saksenaea erythrospora*	UZ1908 15		KU321692	KU321691	–
*Saksenaea erythrospora*	M-891/13		–	KR527486	–
*Saksenaea erythrospora*	CNM-CM8808		MN598586	–	–
*Saksenaea loutrophoriformis*	M-1012/15		LT796164	LT796165	LT796166
*Saksenaea loutrophoriformis*	UTHSC 08-379	ex-type	FR687330	HM776682	HM776693
*Saksenaea longicolla*	C17		MW393835*	MW391838	MW401666
*Saksenaea longicolla*	Sak-06		MW393836*	MW391839	MW401667
*Saksenaea longicolla*	Sak-07, NNIBRFG21789	ex-type	MW393837*	MW391840	MW401668
*Saksenaea longicolla*	Sak-19		MW393838*	MW391841	MW401669
*Saksenaea longicolla*	Sak-21, KACC48577		MK430970*	MW391842	MW401670
*Saksenaea oblongispora*	CBS 133.90	ex-type	NR_137569	NG_057868	HM776687
*Saksenaea trapezispora*	UTHSC DI 15-1, CBS 141687	ex-type	NR_147690	LT607407	LT607408
*Saksenaea trapezispora*	isolate 1855	–	MK321959	–	–
*Saksenaea vasiformis*	AJ1-1		MH059541	–	–
*Saksenaea vasiformis*	ATCC 28740		FR687322	HM776674	HM776685
*Saksenaea vasiformis*	ATCC 60625		FR687323	HM776675	HM776686
*Saksenaea vasiformis*	CNRMA 05.1337		EU182902	–	–
*Saksenaea vasiformis*	CNRMA 07.577		EU644757	EU644756	–
*Saksenaea vasiformis*	CNRMA 08 1143		KP132600	–	–
*Saksenaea vasiformis*	CNRMA F/9-83		FR687325	HM776677	HM776688
*Saksenaea vasiformis*	F5		MF187627	–	–
*Saksenaea vasiformis*	FMR 10131		FR687326	HM776678	HM776689
*Saksenaea vasiformis*	NRRL 2443	ex-type	FR687327	HM776679	AF157291
*Saksenaea vasiformis*	PHF-MC2		MK346253	–	–
*Saksenaea vasiformis*	PHF-MC200		MK501620	–	–
*Saksenaea vasiformis*	PHF-MC201		MK499472	–	–
*Saksenaea vasiformis*	PWQ2338		KP132601	–	–
*Saksenaea vasiformis*	TN254AU15		KU314816	–	–
*Saksenaea vasiformis*	UTHSC 09-528		FR687329	HM776681	HM776692
*Saksenaea vasiformis*	UTHSC R-2974		FR687332	HM776684	HM776695
*Saksenaea vasiformis*	isolate clinical		MG754348	–	–
*Saksenaea vasiformis*	F-375		OQ299499	–	–
*Saksenaea vasiformis*	M5		–	MZ695844	-
*Saksenaea vasiformis*	NCCPF:111021		MZ619078	OK175672	–
*Saksenaea vasiformis*	NCCPF:111022		OP648298	–	–
*Saksenaea vasiformis*	ZESA8		MW340912	–	-
*Saksenaea* sp.	Cesf-21		MK775965	–	–
*Saksenaea* sp.	F2Sc		OL981347	–	–
*Saksenaea* sp.	MF11		MT606215	–	–

Bold accession numbers indicated the sequences were determined in this study

^*^ITS2 region (177–189 bp) is only available in these sequences

For the endofungal bacterium, 16S rRNA gene sequences of *Burkholderiaceae*-related endobacteria and other sequences related to the family *Burkholderiaceae* were obtained from GenBank with the accession numbers shown beside each taxon name in Additional file 5: Fig. S4. These sequences and 16S rRNA gene sequences of the endofungal bacterium obtained from fungal mycelia and pure cultures as DNA templates were aligned, and poorly aligned positions in the alignment block were removed automatically and manually as described above. Subsequently, the alignment block (1329 positions) was used for model selection for ML phylogeny using MEGA 6.06. The ML phylogenetic analysis was performed using RAxML version 8.1.5 with a 1,000 bootstrap replicates. The nucleotide substitution model was set as the GTRGAMMAI model selected in MEGA 6.06.

## Results

### Identity of the fungal host

**Fig. 2:**
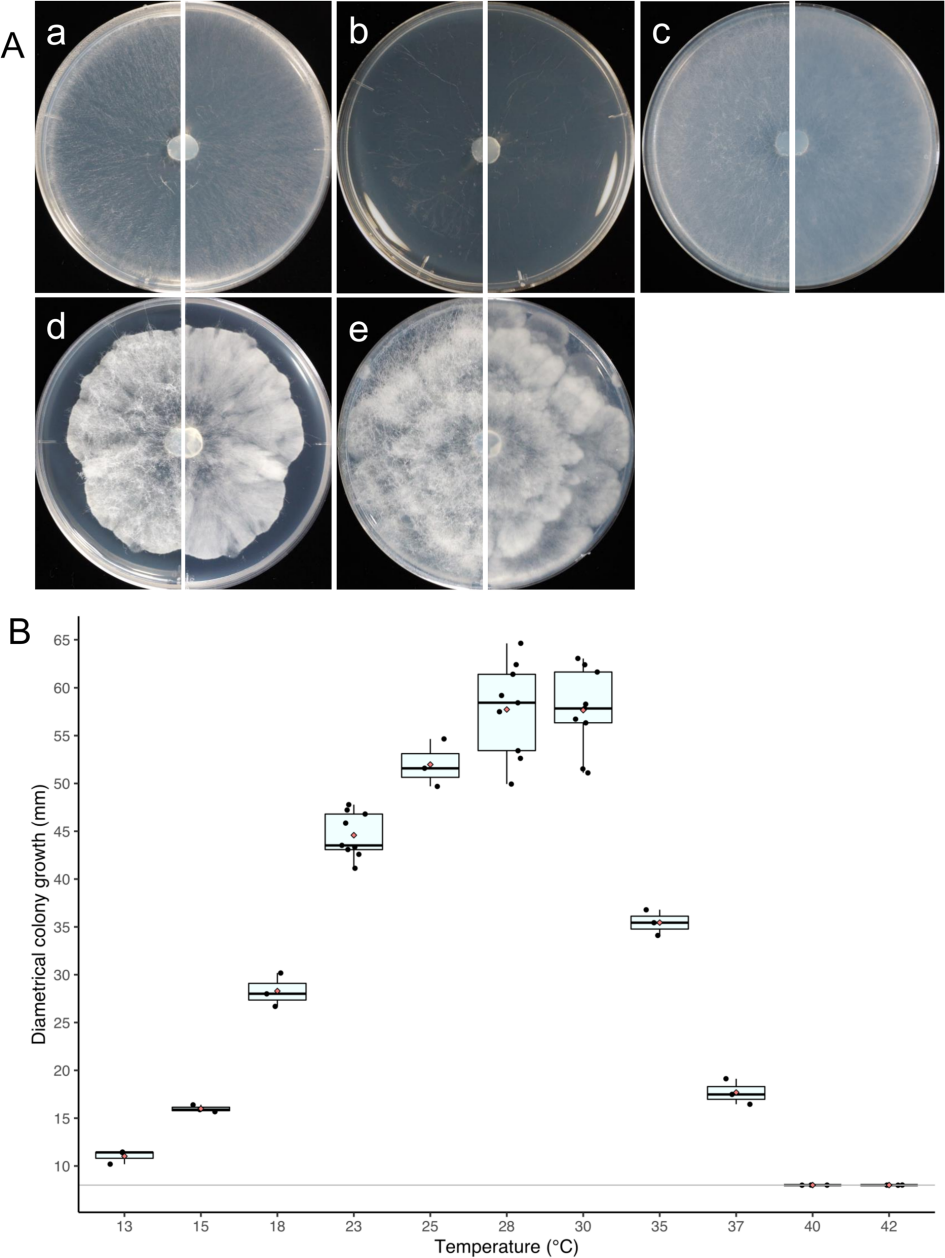
Colony growth of *Saksenaea boninensis* Sak4. **A**: Colony appearance of *Saksenaea boninensis* Sak4 incubated for 4 d at 30 °C on different media [CMA **a**, CZA **b**, _LC_A **c**, MEA **d**, and PDA **e**]. Left and right photos of each medium were front and reverse sides of colonies, respectively. **B**: Diametrical colony growth of *Saksenaea boninensis* Sak4 incubated on CZA for 3 d at different temperatures. Optimum growth was observed around 28–30 °C. No growth was observed above 40 °C. Three replicates (N = 3) were measured for each temperature except for 23, 28, and 30 °C (N = 9). Pinkish diamonds indicate the mean value

The representative isolate Sak4 obtained from litter collected from Haha-jima Island located in the Ogasawara Islands showed the unique morphology of sporangium having a long neck (Fig. 1), which is one of the characteristic morphologies of the genus *Saksenaea* in *Mucorales* (Saksena 1953). Seven species (*S. dorisiae*, *S. erythrospora*, *S. longicolla*, *S. loutrophoriformis*, *S. oblongispora*, *S. trapezispora*, and *S. vasiformis*) are currently recognized in this genus (Alvarez et al. 2010; Crous et al. 2016; Crous et al. 2017; Labuda et al. 2019; Nam et al. 2021; Saksena 1953). The genus *Saksenaea* is also known as one of the thermotolerant fungi in *Mucorales* and the maximum growth temperature exceeds over 40 °C in five species except for *S. dorisiae* and *S. trapezispora* (Alvarez et al. 2010; Baijal 1965; Crous et al. 2017; Nam et al. 2021). The growth range of the representative isolate Sak4 was determined as 13–37 °C on CZA (Fig. 2). The growth at the threshold low and high temperatures (15 and 37 °C, respectively) of the genus was *ca.* 6.0 mm/d (*ca.* 22 mm after 4 d on CZA) and *ca.* 3.7 mm/d (*ca.* 20 mm after 4 d on CZA), respectively. The lower maximum growth temperature (less than 40 °C) was similar to that of *S. dorisiae* and *S. trapezispora* (Crous et al. 2016; Labuda et al. 2019). The minimum growth temperature of the representative isolate Sak4 (13 °C) was closer to that of *S. dorisiae* (12 °C) than that of *S. trapezispora* (15 °C). The growth of the representative isolate Sak4 at the higher temperature (37 °C) was 19–23 mm on CZA for 4 d, which was slightly slower than that of *S. dorisiae* (25–30 mm). Detailed morphological comparisons among the representative isolate Sak4 and these physiologically related species showed that the isolate closely resembled these two species morphologically. However, the morphologies can be distinguished from those of *S. trapezispora* by the longer sporangium and neck (Sak4: 59.6–193.9 µm, *S. trapezispora*: 50–140 µm), and ellipsoidal to cylindrical sporangiospores (Sak4: 4.6–9.7 × 2.7–5.6 µm, *S. trapezispora*: 5.5–7.5 × 3.5–4 µm). The morphologies can be distinguished from those of *S. dorisiae* by having the longer neck (Sak4: 38.0–165.1 µm, *S. dorisiae*: 70–100 µm) and the slightly longer and wider sporangiospores (Sak4: 4.6–9.7 × 2.7–5.6 µm, *S. dorisiae*: 5.0–5.5 × 2.5–3.0 µm).

**Fig. 3:**
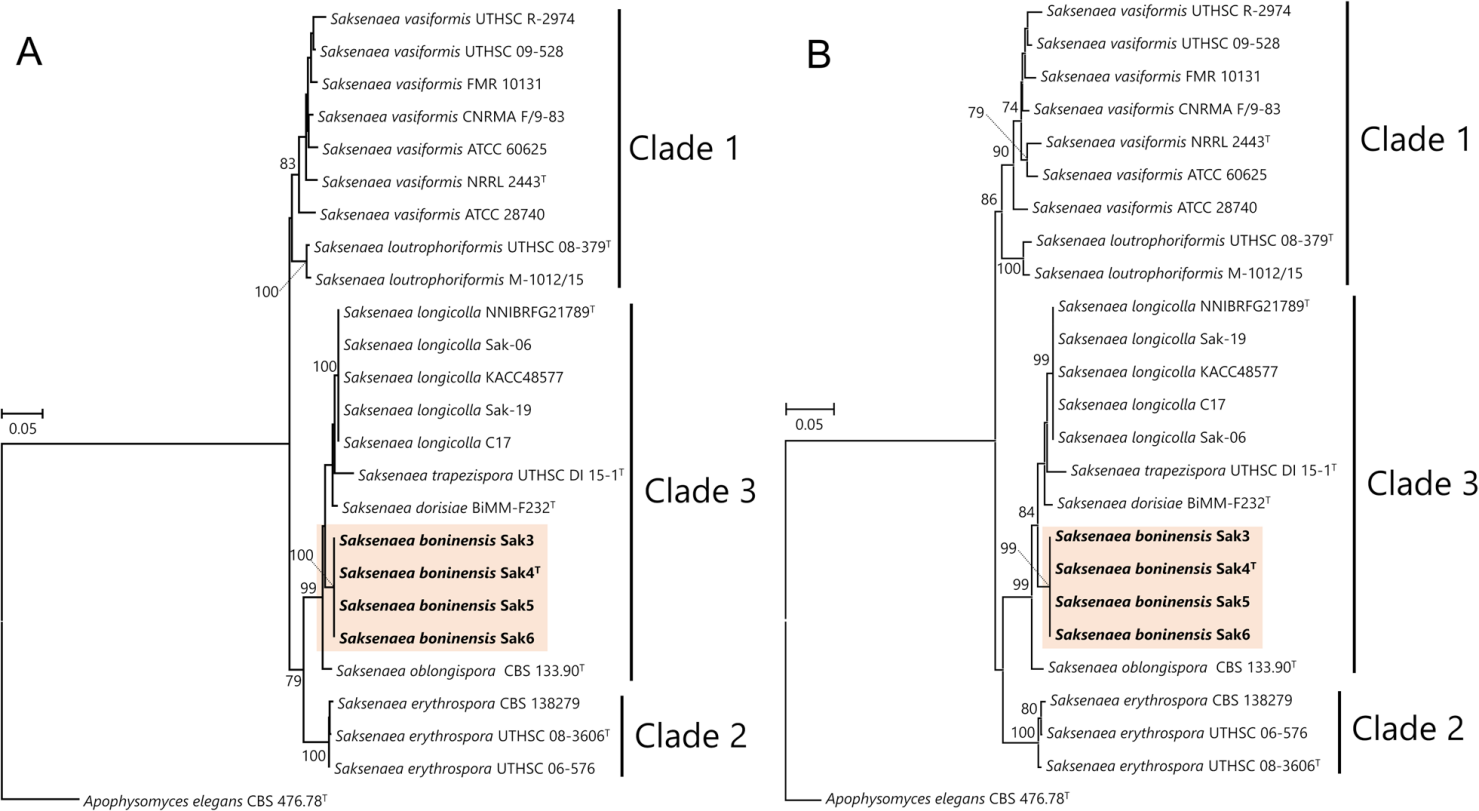
Maximum likelihood (ML) phylogenetic trees of *Saksenaea* spp. based on the concatenated sequences. **A**: dataset 1 (1,276 positions in total) consisted of the ITS2 region (168 positions), the partial LSU region (637 positions), and the partial *tef1* gene (471 positions). The value of the log likelihood was − 3633.090024. **B**: dataset 2 (1,662 positions in total) consisted of the ITS1-5.8S-ITS2 region (554 positions), the partial LSU region (637 positions), and the partial *tef1* gene (471 positions). The value of the log likelihood was − 5163.803276. Bootstrap values ≥ 70% are shown at nodes. *Apophysomyces elegans* was used as the outgroup. *Saksenaea* spp. were clustered into three clades as defined by Alvarez et al. (2010). “T” beside each strain name indicates the strains as ex-type strains

The Maximum likelihood (ML) phylogenetic trees showed that the isolates were phylogenetically identical to each other (Fig. 3, Additional file 2–4: Fig. S1–S3), indicating the isolates probably derived from a single individual. In the phylogenetic trees using concatenated datasets, the differences of the datasets were not affected to tree topologies and the isolates were located in a highly supported clade, “clade 3” defined by Alvarez et al. (2010), containing isolates of *S. dorisiae*, *S. longicolla*, *S. oblongispora*, and *S. trapezispora* (Fig. 3A and B). This result suggests that the present isolates were phylogenetically close to these four species. The pairwise distances of the ITS2, which was more variable region among the four regions calculated in this study, showed that the nucleotide divergences among “clade 3” containing the representative isolate Sak4 and four species were ranged from 0.6 to 5.5%, and the closest value was found in *S. dorisiae* and *S. longicolla* as 0.6% (Additional file 1: Table S1). While the nucleotide divergences between the representative isolate Sak4 and each species in the “clade 3” were more than 4.9% (4.9–5.5%), suggesting the representative isolate Sak4 was distinct from these four species phylogenetically (Additional file 1: Table S1). Among these four phylogenetically related species, the representative isolate Sak4 was physiologically close to *S. dorisiae* and *S. trapezispora*, but it can be distinguished from these two species morphologically as described above. The remaining two species were physiologically different from the representative isolate Sak4 in terms of growth temperature preferences such as the higher and narrow optimum temperatures (Sak4: 28–30 °C, *S. longicolla*: 25–37 °C, *S. oblongispora*: 25 °C), and the lower maximum growth temperatures (Sak4: 37 °C, *S. longicolla*: 40 °C, *S. oblongispora*: the exact value was not indicated, but could be placed at least between 37 and 42 °C). Since *S. oblongispora* has a shorter neck (60–90 µm), similar to those of *S. dorisiae* and *S. trapezispora*, this species can be morphologically distinguished from the representative isolate Sak4. While the ranges of the length of the neck and the size of sporangiospores of *S. longicolla* were overlapped to those of the representative isolate Sak4; however, the morphologies of the representative isolate Sak4 can be distinguished from those of *S. longicolla* by having more longer neck (Sak4: 59.6–193.9 µm, *S. longicolla*: 35–151 µm) and the slightly longer and wider sporangiospores (Sak4: 4.6–9.7 × 2.7–5.6, *S. longicolla*: 5.0–7.9 × 2.5–3.9 µm). Therefore, the representative isolate Sak4 was morphologically, physiologically, and phylogenetically distinguishable from known species and proposed as a new species, *S. boninensis*.

Of note, the ML phylogenetic tree using ITS1-5.8S-ITS2 including most of the currently registered sequences in GenBank showed many sequences registered such as “*S. vasiformis*” were not clustered into the clade containing the ex-type strain of *S. vasiformis* (Additional file 2: Fig. S1). The isolation sources of such “incorrect” sequences were mostly obtained from clinical sources. While the sequences associated with species in the “clade 3”, including *S. boninensis*, tend to be more likely to have been isolated from non-animal related isolation sources, especially the sequence of “*S. vasiformis*” strain ZESA8 (MW340912) isolated from the root of *Prunus armeniaca* in Iraq was the most closely related to that of *S. boninensis* (Additional file 2: Fig. S1).

### Taxonomy

***Saksenaea boninensis*** Y. Takash., K. Narisawa, **sp. nov.**

MycoBank no.: MB 843122.

Figures 1, 2.

*Diagnosis*: The longer neck of sporangia, longer and wider sporangiospores, and slightly more sensitive growth at the threshold high temperature (37 °C) are distinctive characters of this species compared with other known *Saksenaea* spp.

*Type*: **Japan**: *Tokyo*: Ogasawara Islands, Haha-jima Island, Ogasawara-mura, Koromodate, near the Kitako port (north port), isolated from a culture plate (_LC_A medium) directly inoculated with litter, 1 Dec 2018, *Y. Takashima* (KPM-NC0028612, dried fungal material on CZA – holotype; Sak4 = MAFF 247844 = JCM 39173 = NBRC 114970 = CBS 147591 – ex-holotype cultures).

*Gene sequences ex-holotype*: MK757863 (ITS), MK757859 (LSU), LC474957 (*tef1*).

*Etymology*: *boninensis*, referring to the Bonin Islands (Ogasawara Islands), the geographic origin of the type.

*Description: Colonies* on CZA fast growing, filling the 90-mm-diam Petri dish after 4 d of incubation (*ca.* 20 mm/d) at the optimum growth temperatures (28 and 30 °C), hyaline, with scarce aerial mycelia, reverse concolorous. The minimum growth observed at 13 °C (*ca.* 3.3 mm/d, *ca.* 15 mm after 4 d on CZA). No growth observed at 38 °C. Colonies on CMA, _LC_A, MEA, PDA at 30 °C showing whitish and floccose with aerial mycelia, lobed pattern observed on PDA, reverse concolorous. Faster colony growth at 30 °C observed on CMA, _LC_A, PDA (*ca.* 25–30 mm/d) than that on CZA and MEA (*ca.* 20 mm/d). Sporulation at 30 °C observed on CMA, CZA, and _LC_A within 14 d, but not on MEA and PDA. Sporulation on CZA observed ranging from 18 to 30 °C, and more abundant at 23 °C. *Hyphae* 1.9–10.3 (Mean ± SD = 4.7 ± 2.0) µm wide. *Sporangia* generally single, rarely two sporangia occurred on a rhizoid, erect, developed at the end of a hyphal branch basally narrowed in a stalk bearing dichotomously branched rhizoids, flask-shaped with a brownish spherical venter surmounted by a brownish long neck, with a distinct dome-shaped columella; rhizoids hyaline, 2.9–5.8 (Mean ± SD = 4.3 ± 0.6) µm wide; stalk brownish, 4.9–13.3 (Mean ± SD = 8.2 ± 2.2) × 44.9–89.4 (Mean ± SD = 68.0 ± 11.2) µm; venter 18.8–41.3 (Mean ± SD = 28.9 ± 5.7) × 21.4–50.4 (Mean ± SD = 34.4 ± 6.8) µm; neck 5.5–11.9 (Mean ± SD = 8.9 ± 1.3) × 38.0–165.1 (Mean ± SD = 93.2 ± 26.5) µm [venter + neck: 59.6–193.9 (Mean ± SD = 124.0 ± 31.2) µm long], apex of the neck slightly broader, 8.2–15.2 (Mean ± SD = 12.0 ± 1.8) µm in diam, closed with a mucilaginous plug, which is gradually dissolved when mature. Surface of brownish parts of sporangia (stalk, venter, and neck) asperulate, ornamented with fine spines, dissolved in lactic acid within 12 h. Sporangiospores hyaline, ellipsoidal to cylindrical, 4.6–9.7 (Mean ± SD = 6.8 ± 1.1) × 2.7–5.6 (Mean ± SD = 3.5 ± 0.5) µm [Q (quotients of spore length and width) = 1.4–2.7, Q_m_ (the mean Q value) = 1.9]. *Zygospores* unknown.

*Note*: A *Burkholderiaceae*-related endobacterium has been associated with isolates of this species since the isolates were obtained. However, the morphological differences between isolates with/without the endofungal bacterium, such as sporulation of sporangiospores observed in *R. microsporus* (Lackner et al. 2011), were not examined in this study.

### Discovery of new BRE lineage from isolates of *Saksenaea*

**Fig. 4:**
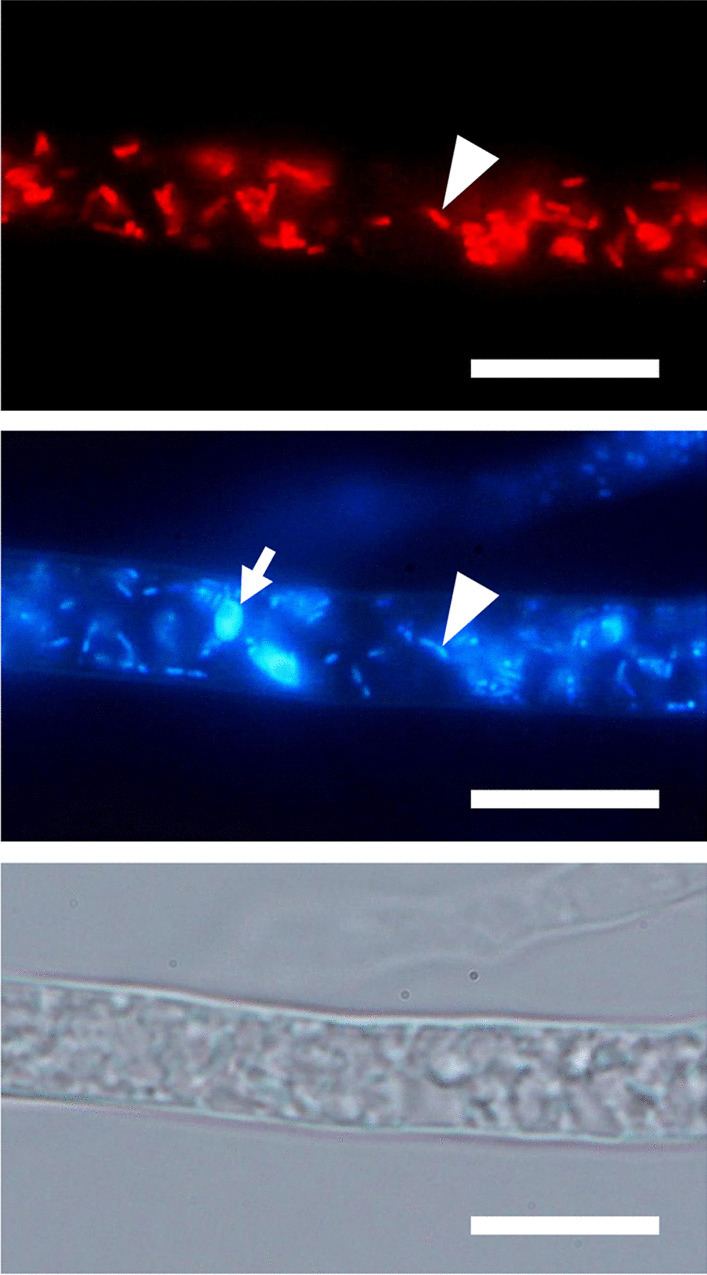
FISH image of SakBRE associated with *Saksenaea boninensis* Sak4. FISH was performed using the Cy3-labeled (Red) EUB338 probe (top). DAPI (center) and bright field (bottom) images are also shown. A representative host nucleus and bacterial cell within a hypha are indicated by an arrow and arrowhead, respectively. Scale bars: 10 μm

**Fig. 5:**
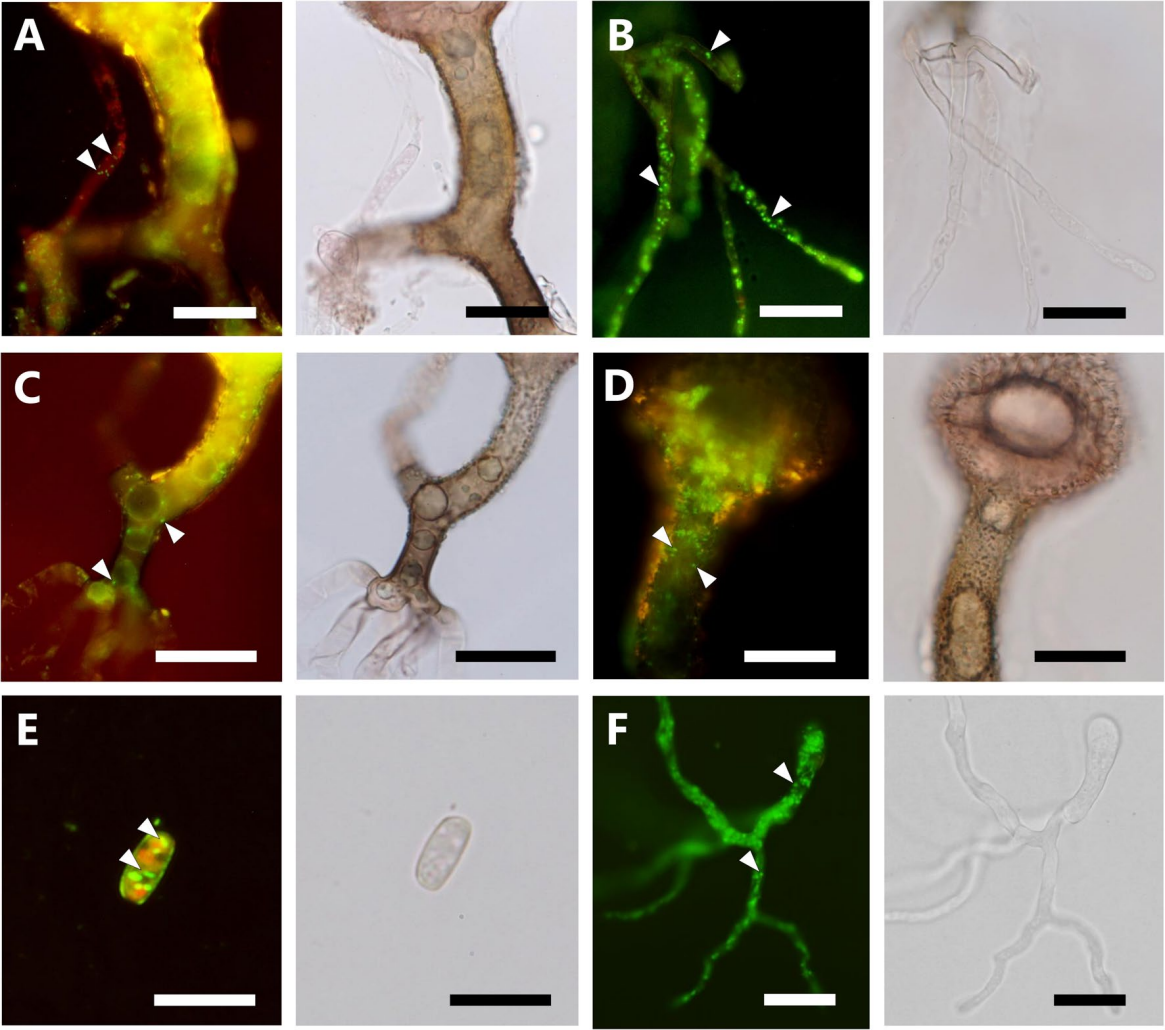
LIVE/DEAD stained fluorescence images of SakBRE associated with *Saksenaea boninensis* Sak4. Bright field images are shown beside each fluorescence image. Bacterial cells are indicated by arrowheads. Rod-shaped endofungal bacterium-like cells were localized within asexual stages such as aerial hypha **A**, rhizoid **B**, stalk **C**, sporangium **D**, sporangiospore **E**, and germinated sporangiospore incubated for 12 h on _LC_A **F**. Scale bars: **A**–**D** 20 μm; **E**, **F** 10 μm

**Fig. 6:**
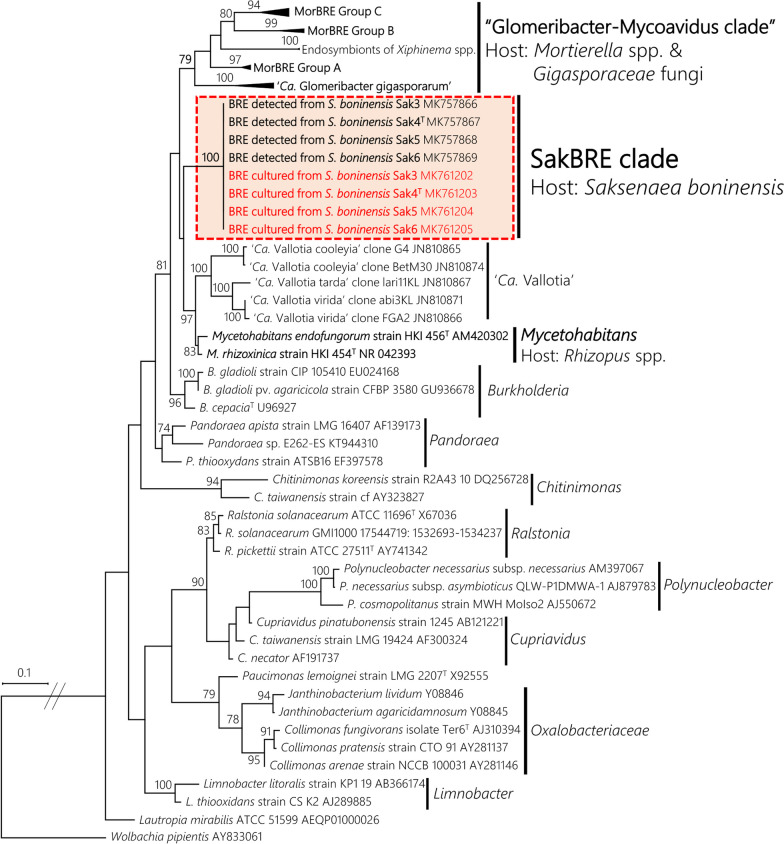
Maximum likelihood (ML) phylogenetic tree of *Burkholderiaceae* based on 16S rRNA gene sequences (1329 positions). Bootstrap values ≥ 70% are shown at nodes. The value of the log likelihood was − 12,214.778642. *Wolbachia pipientis* was used as the outgroup. “T” beside each strain name indicates the strains as ex-type strains. The SakBRE clade consisted of the obtained sequences in this study, indicated by a square with dashed red lines. In the square,16S rRNA gene sequences determined from the DNA templates prepared from BRE-harboring mycelia and pure cultures of the BRE are shown in black and red letters, respectively

As the survey for the endofungal bacteria associated with *Mucoromycota*, all isolates obtained from different sporangia were subjected to PCR amplification of 16S rRNA using the DNA templates prepared from fungal mycelia. Since positive amplification was confirmed in all isolates, fluorescence microscopic observations were conducted to confirm the presence of endofungal bacteria. FISH observation of the representative isolate Sak4 using the Cy3-labeled bacterial universal probe showed the presence of the endofungal bacterium within hypha (Fig. 4). The observation of the endofungal bacterium using the nucleic staining reagent also showed that the endofungal bacterium was present throughout the asexual stage, such as aerial mycelium, rhizoid, stalk, sporangium, sporangiospore, and geminated sporangiospore (Fig. 5). These observations strongly suggested that the amplification of 16S rRNA was derived from the endofungal bacterium, which was vertically transmitted through asexual sporogenesis of the fungal host. The 16S rRNA gene sequences determined from PCR amplicons with fungal mycelia of each isolate were identical to each other. ML phylogeny of the 16S rRNA gene clearly showed that the endofungal bacterium was phylogenetically related to the family *Burkholderiaceae* (Fig. 6, Additional file 5: Fig. S4). However, the phylogenetic position of the endofungal bacterium was not clustered with either “Glomeribacter-Mycoavidus clade” or *Mycetohabitans* spp. (Fig. 6). This result indicates that the endofungal bacterium, which thrived within isolates of *S. boninensis* was the new BRE lineage (hereafter, named “SakBRE”).

### Culturability of SakBRE

**Fig. 7:**
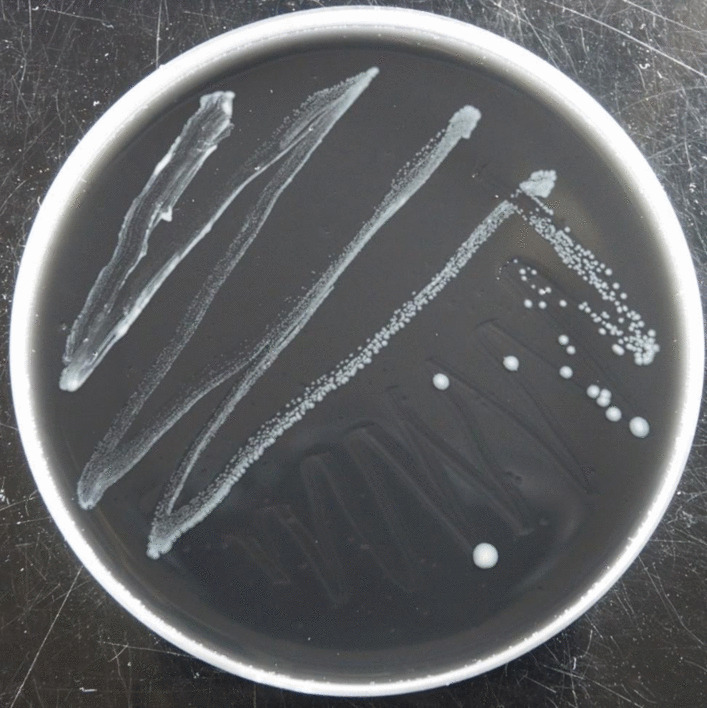
Pure culture of SakBRE grown on BCYEα medium after 14-d incubation at 30 °C

Because *Mycetohabitans* spp. associated with *R. microsporus* are known to be culturable (Partida-Martinez et al. 2007), SakBRE has the potential to be culturable. To attempt bacterial isolation, a filtrated bacterial suspension of SakBRE obtained from each isolate was inoculated onto BCYEα medium. As expected, bacterial growth by visually increasing the bacterial density in the filtrates was confirmed after 3 to 4 d at 30 °C and after 8 d at 23 °C, respectively. The clouded filtrates were streaked onto fresh BCYEα medium and incubated at 30 °C, and then pure cultures were successfully established from single colonies obtained after incubation for at least 5 d (Fig. 7). The 16S rRNA gene sequences obtained from the pure cultures were identical to the sequences detected from fungal mycelia of the host (Fig. 6). We also checked the culturability in several media by streaking the obtained pure cultures on BCYEα medium, LB agar, 1/10 nutrient broth agar, and R2A agar media, and incubating at 30 °C. Bacterial growth was confirmed after at least 5d on BCYEα medium (Fig. 7), while growth was not observed on the other three media even after 14 d.

## Discussion

The number of species of the genus *Saksenaea* has recently increased; however, its diversity is not fully understood. In Japan, the occurrence of *Saksenaea* was very rarely reported, while no clinical cases of mucormycosis caused by *Saksenaea* spp. have been reported (Mori et al. 2011). “*Saksenaea vasiformis*” was only found in soil from the Ryukyu Islands, in Japan, previously (Watanabe 1971; Tubaki et al. 1990). The isolate 69-323 obtained by Watanabe (1971) was identified as “*S. vasiformis*”, and morphologies such as the length of the neck (*ca.* 32.5 µm) and size of sporangiospores (up to 5.0 × 2–2.5 µm) were not similar to the present species (Watanabe 2010). Chien et al. (1992) isolated “*S. vasiformis*” from sources near marine environments such as soil around the seashore in Taiwan and unidentified intertidal driftwoods along the seacoast in Ethiopia. Our isolation of *Saksenaea* from the Kitako port (north port) of Haha-jima Island may indicate that this genus is likely to prefer places with a subtropical oceanic climate in Japan and has an affinity for marine environments as one of its natural habitats. In the present study, the ML phylogenetic tree using ITS1-5.8S-ITS2 showed that *Saksenaea* spp. especially related to the “clade 3” including *S. boninensis* were likely to be found in environmental sources. This result suggested that the known habitats of this genus in non-clinical samples remained to be uncharacterized and emphasized the importance of more isolation of *Saksenaea* spp. from environmental samples.

To date, screenings of endofungal bacteria with broad-ranging fungal hosts at the generic level in *Mucorales* have been conducted in few studies. Schmitt et al. (2008) screened over 300 zygomycete strains other than *R. microsporus* (the exact number of genera used was not shown in the article) and they concluded that endofungal bacteria were limited to detection in *R. microsporus*. On the other hand, Okrasińska et al. (2021) screened 71 strains containing 10 genera in *Mucorales*, and they detected *Mycetohabitans* spp. and *Paraburkholderia* sp. from three strains of *R. microsporus* and one strain of *Mucor moelleri*, respectively. The comprehensive screening of bacteria in 64 strains of two *Rhizopus* spp. resulted in the detection of *Mycetohabitans* spp. and *Burkholderia* sp. (Dolatabadi et al. 2016). In a recent comprehensive study of bacteriomes of fungi, four strains (including two genera, *Mucor* and *Rhizopus*) and eight genomes of *Mucorales* (including eight species assigned in each different genus) were screened by a 16S rRNA gene amplicon sequencing and bioinformatic screening, respectively, and numerous bacterial lineages were detected by both analyses (Robinson et al. 2021). However, only one genus from the *Burkholderiaceae* family (namely *Cupriavidus*) was listed as symbiont of *Mucor* sp. in study by Robinson et al. (2021). These screening results suggest that the bacterial lineage regarded as BRE, strictly comprised of endofungal bacteria such as *Mycetohabitans*, are only detected from *Rhizopus* spp. Even though the genus *Saksenaea* is known to be thermotolerant, similar to *Rhizopus*, representatives of this genus are completely overlooked as fungal hosts. Therefore, the finding of the phylogenetically distinct lineage, SakBRE, from the genus *Saksenaea* shown in the present study is notable. The origin and evolution of BRE associated with *Mucoromycota* remain unclear. Bonfante and Desirò (2017) proposed two hypothetical scenarios of bacterial invasion (early vs. late) on the basis of the origin of the associations between BRE and *Mucoromycota*. Our discovery of SakBRE may support the late bacterial invasion scenario of BRE among members of *Mucoromycota*. Our results expand known hosts of BRE in *Mucorales* into the two genera *Rhizopus* and *Saksenaea*, which are classified as different fungal families (*Rhizopodaceae* and *Saksenaeaceae*) and phylogenetically distinct (Hoffmann et al. 2013). This distinct host range adds to the enigma of the origin and evolution of BRE associated with *Mucorales*. Further studies such as a comparative genomics study of SakBRE and *Mycetohabitans* spp. are suggested by this study, which will add new insight into whether these BRE have a single common evolutionary ancestor.

Currently, four BRE species including two species each of the genera *Mycoavidus* and *Mycetohabitans*, respectively, are known to be culturable (Partida-Martinez et al. 2007; Ohshima et al. 2016; Guo et al. 2020). *Mycoavidus* spp. require cysteine for growth and can only be cultured on BCYEα medium (Ohshima et al. 2016; Guo et al. 2020). For its isolation, incubation for 7 d at 30 °C and 30 d at 23 °C is required for *M. cysteinexigens* B1-EB^T^ and *Mycoavidus* sp. B2-EB on the medium, respectively (Ohshima et al. 2016; Guo et al. 2020). On the other hand, the growth of *Mycetohabitans* spp. was faster than that of *Mycoavidus* spp. and these can be cultured within 2–3 days (Rohm et al. 2010) or several days (Scherlach et al. 2006) on conventionally used agar media such as nutrient agar, LB agar, tryptic soy agar, and PDA (Partida-Martinez and Hertweck 2005; Scherlach et al. 2006; Partida-Martinez et al. 2007; Rohm et al. 2010). In comparison with these incubation conditions of BRE, the growth of SakBRE in the isolation step (3 to 4 d within filtrates and 5 d on plate at 30 °C) is faster than that of *M. cysteinexigens* on BCYEα medium but is limited and slower than that of *Mycetohabitans* spp. on the other media. Further analysis such as whole-genome sequencing of SakBRE will be needed to clarify whether the fastidious character of SakBRE comes from a nutrient deficiency or other factors such as hydrogen peroxide generated within media, which is known to be decomposed by activated charcoal in BCYEα medium (Hoffman et al. 1983).

Mucormycosis has received much attention as “Black fungi” due to the increasing number of infections among patients affected by COVID-19 (Chavda and Apostolopoulos 2021; Singh et al. 2021). Thermophilic or thermotolerant mucoralean fungi including the eleven genera: *Actinomucor*, *Apophysomyces*, *Cokeromyces*, *Cunninghamella*, *Lichtheimia*, *Mucor*, *Rhizomucor*, *Rhizopus*, *Saksenaea*, *Syncephalastrum*, and *Thamnostylum* are known as causative agents of mucormycosis (Ajello et al. 1976; Chakrabarti and Singh 2014; Chander et al. 2018; Roden et al. 2005). In the present study, most species in “clade 3” including *S. boninensis* were isolated from non-animal substrates (fungi, litter, plant, soil, and water) and sensitive to high temperatures except for *S. oblongispora* (Fig. 3, Additional file 2: Fig. S1), and there have been no clinical cases in humans to date (Alvarez et al. 2010; Crous et al. 2016; Labuda et al. 2019; Nam et al. 2021). However, it remains a possibility that this species is an animal pathogen because its close relative, *S. trapezispora*, was isolated from the knee wound of a soldier (Crous et al. 2016).

*Mycetohabitans* spp. were previously detected from clinical isolates of *Rhizopus* spp. (Ibrahim et al. 2008; Partida-Martinez et al. 2008) and directly isolated from clinical specimens (Gee et al. 2011). The contributions of rhizoxin-producing *Mycetohabitans* spp. to *Rhizopus* virulence were debated previously (Ibrahim et al. 2008; Partida-Martinez et al. 2008), and it was demonstrated that such endofungal bacteria are not essential for *Rhizopus* infection by comparing *Mycetohabitans*-harboring and -free strains in infection models, such as human endothelial cell, fly, and mouse models. On the other hand, recently in *R. microsporus*, *Ralstonia pickettii* was discovered as an endofungal bacterium (Itabangi et al. 2022). This bacterium is not capable of synthesizing rhizoxin but is required for *Rhizopus* virulence in both zebrafish and mouse models and reduces the sensitivity of the fungal host to the antifungal treatment Amphotericin B (Itabangi et al. 2022). Although it is still unclear whether SakBRE can produce harmful toxins and is involved in host virulence in mucormycosis, our results emphasize the importance of careful screening of endofungal bacteria in clinical isolates, especially *Rhizopus* and *Saksenaea* spp. and the related genus *Apophysomyces*, and other thermotolerant mucoralean environmental isolates that are potentially causative agents of mucormycosis should be monitored.

## Conclusion

In the present study, we described a new species, *Saksenaea boninensis*, which was morphologically, physiology, and phylogenetically distinguishable from known species. In Japan, species of the genus *Saksenaea* was firstly isolated from the Ogasawara (Bonin) Islands other than the Ryukyu Islands. We showed the presence of a new BRE lineage (SakBRE) within the hyphae in this species, the vertical transmission through asexual sporogenesis by SakBRE, and the growth of SakBRE on the artificial media which was less fastidious than *Mycoavidus* spp. The discovery of new culturable BRE from *Mucorales* as well as *Rhizopus* spp. will add new insight into the evolutional origin of mucoralean fungus-BRE associations and emphasize the need to pay more attention to endofungal bacteria potentially associated with isolates of thermotolerant mucoralean fungi causing mucormycosis.

### Supplementary Information

**Additional file 1 Table S1.** Pairwise distances of the nucleotide sequences (**A**: ITS2, **B**: ITS1-5.8S-ITS2, **C**: LSU, and **D**: *tef1*) of the ex-type strains of *Saksenaea *spp. The clades of each species were inferred from the phylogenetic tree using the concatenated dataset containing ITS1-5.8S-ITS2, LSU, and *tef1* gene sequences.

**Additional file 2 Fig. S1.** Maximum likelihood (ML) phylogenetic tree of *Saksenaea* spp. based on the ITS1-5.8S-ITS2 region (533 positions). Bootstrap values ≥50% are shown at nodes. The value of the log likelihood was − 2696.671725. *Apophysomyces elegans* was used as the outgroup. “T” beside each strain name indicates the strains as ex-type strains. The taxa names are shown in red if the species name registered in GenBank were not included in the clade containing the ex-type strain of each species. Countries where strains were isolated and isolation sources of each strain were shown following to strain names except for *S. boninensis*. Taxa names highlighted by blue indicate isolation sources were non-animal related materials such as fungi, litter, plant, soil, and water.

**Additional file 3 Fig. S2.** Maximum likelihood (ML) phylogenetic tree of *Saksenaea* spp. based on the partial LSU region (602 positions). Bootstrap values ≥50% are shown at nodes. The value of the log likelihood was − 1508.912359. *Apophysomyces elegans* was used as the outgroup. “T” beside each strain name indicates the strains as ex-type strains.

**Additional file 4 Fig. S3.** Maximum likelihood (ML) phylogenetic tree of *Saksenaea* spp. based on the partial tef1 gene (471 positions). Bootstrap values ≥50% are shown at nodes. The value of the log likelihood was − 1008.331410. *Apophysomyces elegans* was used as the outgroup. “T” beside each strain name indicates the strains as ex-type strains.

**Additional file 5 Fig. S4.** The full version of Figure 6 shown in this study.

## Data Availability

Nucleotide sequences of the fungal isolates generated during this study were deposited in GenBank [MK757862–MK757865 (ITS), MK757858–MK757861 (LSU), LC474956–LC474959 (*tef1*)]. Nucleotide sequences of the 16S rRNA gene detected from fungal mycelia of each isolate and from pure cultures were deposited in GenBank (MK757866–MK757869 and MK761202–MK761205, respectively). Multiple sequence alignments for the phylogenetic analyses of *Saksenaea* spp. and the family *Burkholderiaceae* generated during this study are available in the Zenodo repository, https://doi.org/10.5281/zenodo.6303648.

## Abbreviations

1/2 CMMY: Half-strength Corn Meal-Malt-Yeast Agar medium
BCYEα: Buffered charcoal yeast extract with 0.1% α-ketoglutalate
BRE: *Burkholderiaceae*-related endobacteria
CMA: Corn Meal Agar medium
CZA: Czapek-Dox Agar medium
ITS: Internal transcribed spacer region
LB: Luria–Bertani
LSU: Large subunit (28S) rRNA gene region
MEA: Malt Extract Agar medium
ML: Maximum-likelihood
PDA: Potato Dextrose Agar medium
SakBRE: A BRE lineage thrived within isolates of *Saksenaea boninensis*
*tef1*: Translation elongation factor 1-α gene
