# Supplementary figures and images for: Detection and isolation of a new member of Burkholderiaceae-related endofungal bacteria from Saksenaea boninensis sp. nov., a new thermotolerant fungus in Mucorales
Source: IMA Fungus. 2023 Nov 23;14:24. doi: 10.1186/s43008-023-00129-2 (PMC10666400; doi:10.1186/s43008-023-00129-2)

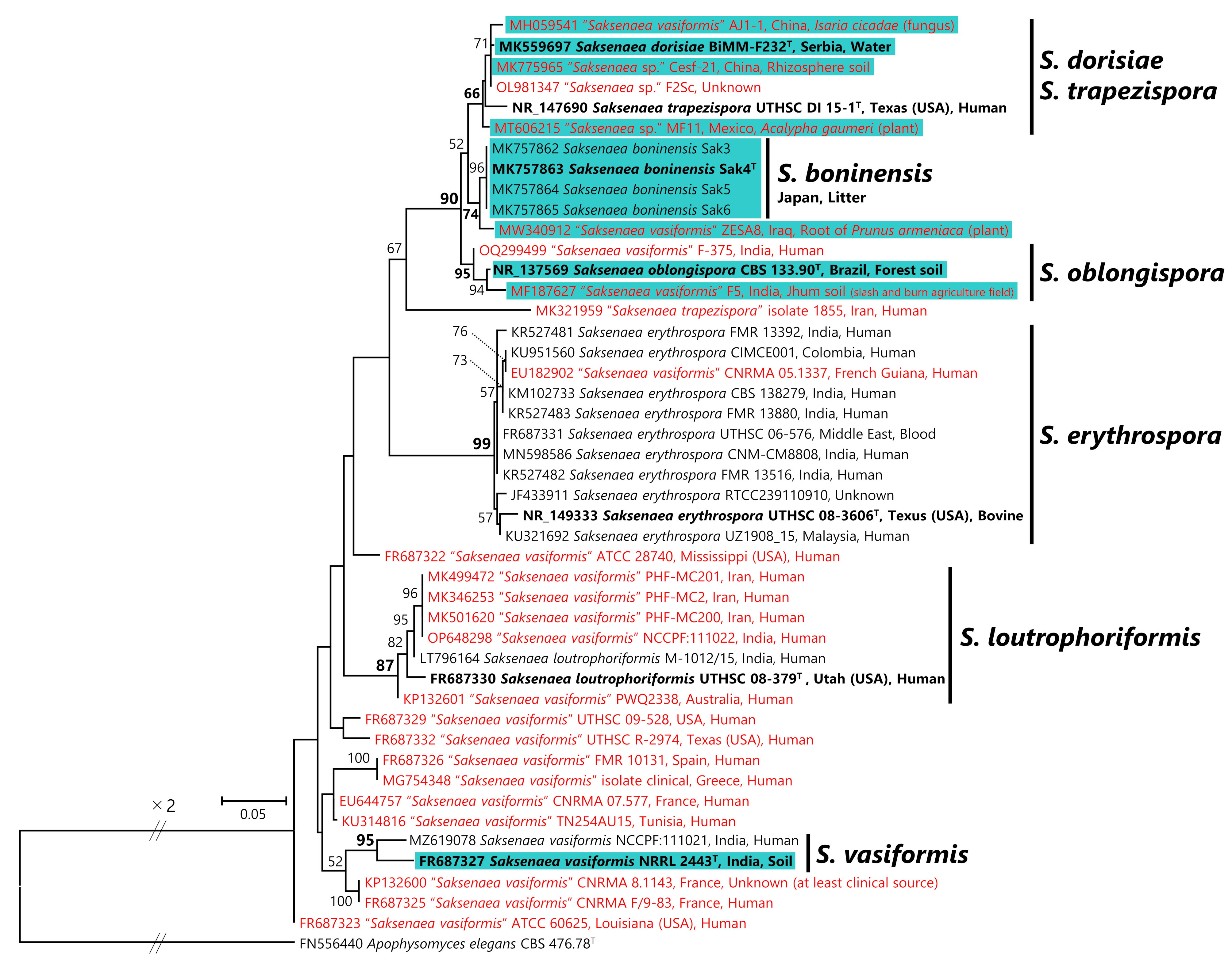

Supplement: Supplementary file 2 — Additional file 2 Fig. S1. Maximum likelihood (ML) phylogenetic tree of Saksenaea spp. based on the ITS1-5.8S-ITS2 region (533 positions). Bootstrap values ≥50% are shown at nodes. The value of the log likelihood was − 2696.671725. Apophysomyces elegans was used as the outgroup. “T” beside each strain name indicates the strains as ex-type strains. The taxa names are shown in red if the species name registered in GenBank were not included in the clade containing the ex-type strain of each species. Countries where strains were isolated and isolation sources of each strain were shown following to strain names except for S. boninensis. Taxa names highlighted by blue indicate isolation sources were non-animal related materials such as fungi, litter, plant, soil, and water. [file 43008_2023_129_MOESM2_ESM.tiff]

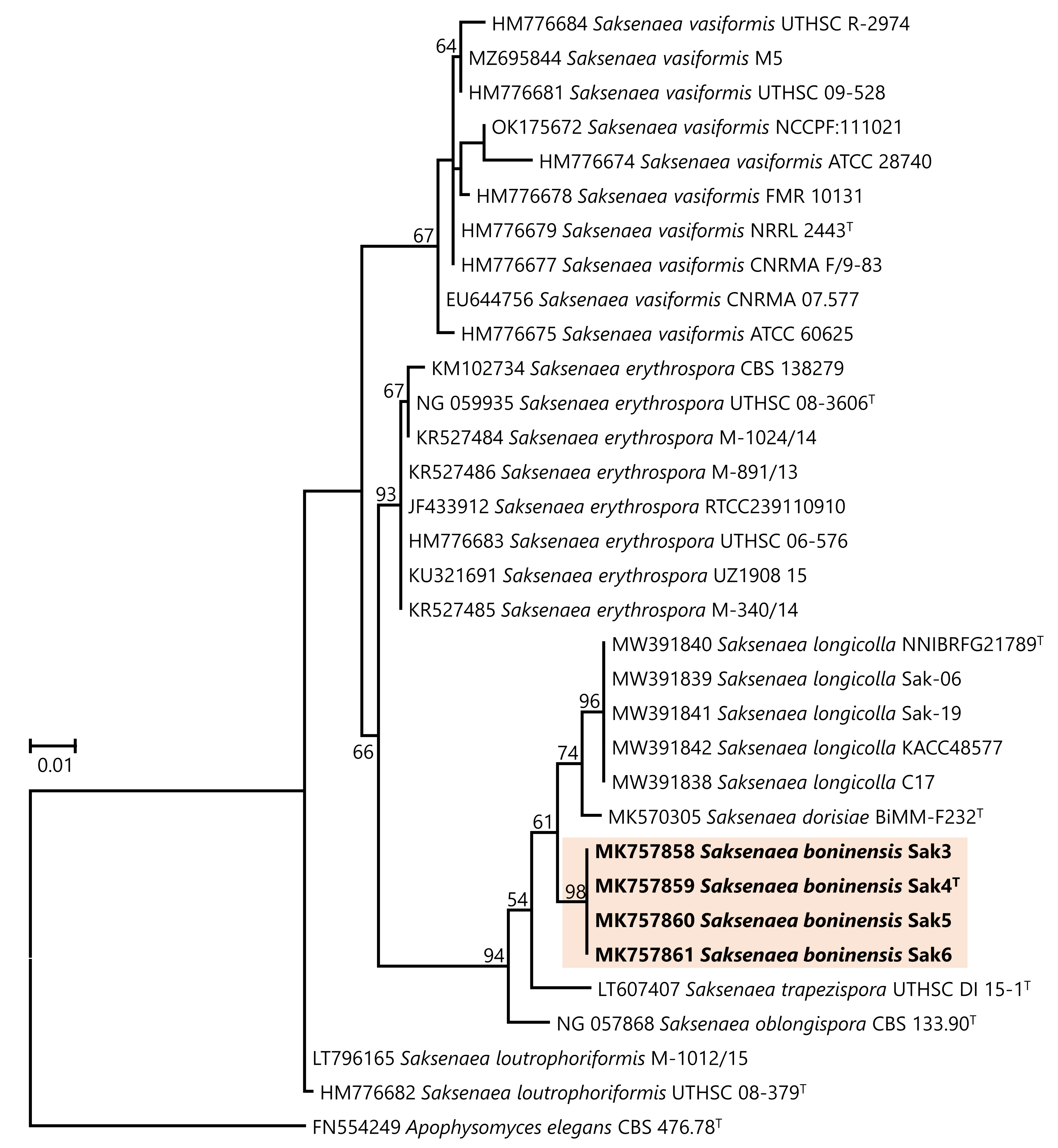

Supplement: Supplementary file 3 — Additional file 3 Fig. S2. Maximum likelihood (ML) phylogenetic tree of Saksenaea spp. based on the partial LSU region (602 positions). Bootstrap values ≥50% are shown at nodes. The value of the log likelihood was − 1508.912359. Apophysomyces elegans was used as the outgroup. “T” beside each strain name indicates the strains as ex-type strains. [file 43008_2023_129_MOESM3_ESM.tiff]

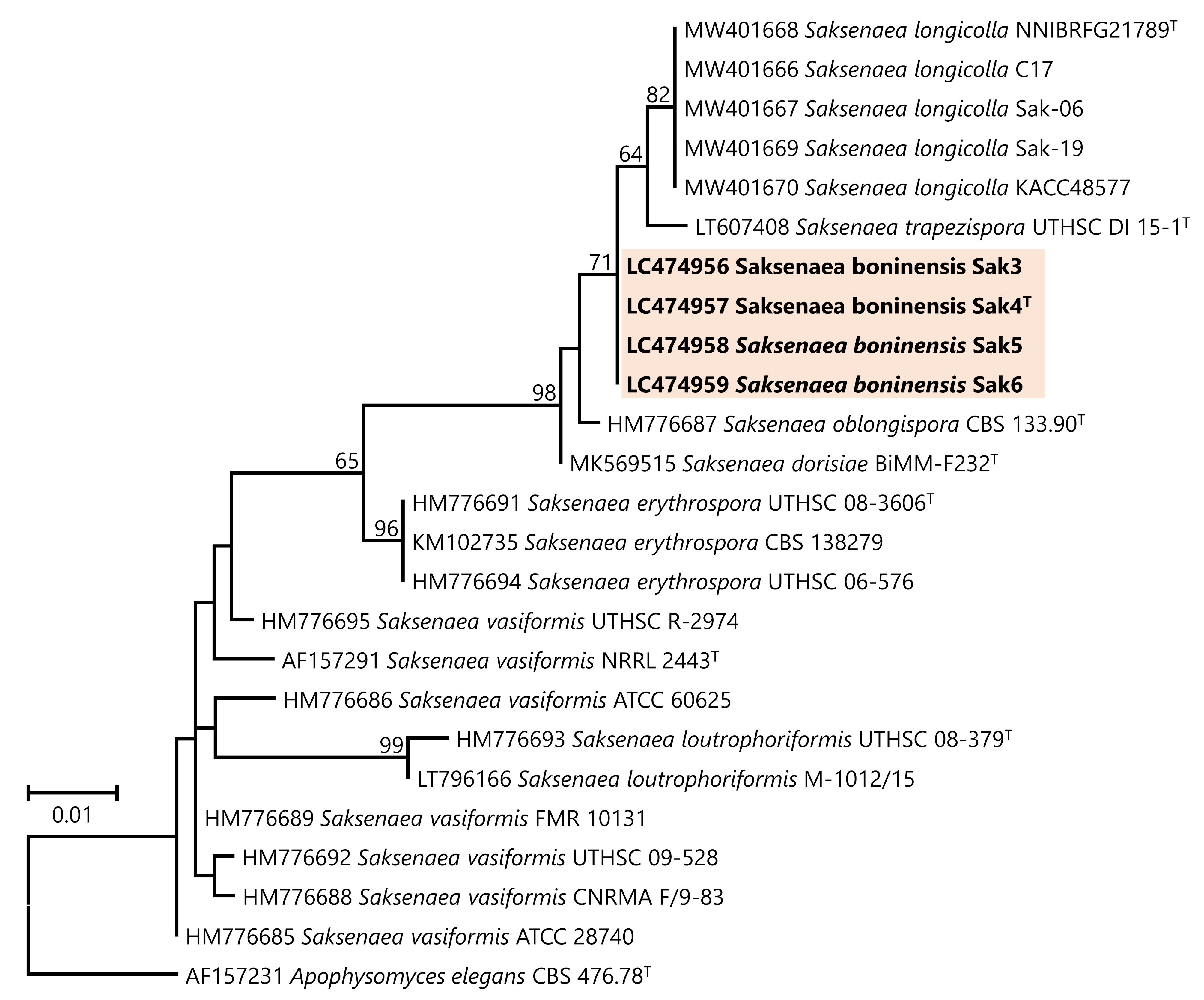

Supplement: Supplementary file 4 — Additional file 4 Fig. S3. Maximum likelihood (ML) phylogenetic tree of Saksenaea spp. based on the partial tef1 gene (471 positions). Bootstrap values ≥50% are shown at nodes. The value of the log likelihood was − 1008.331410. Apophysomyces elegans was used as the outgroup. “T” beside each strain name indicates the strains as ex-type strains. [file 43008_2023_129_MOESM4_ESM.tiff]

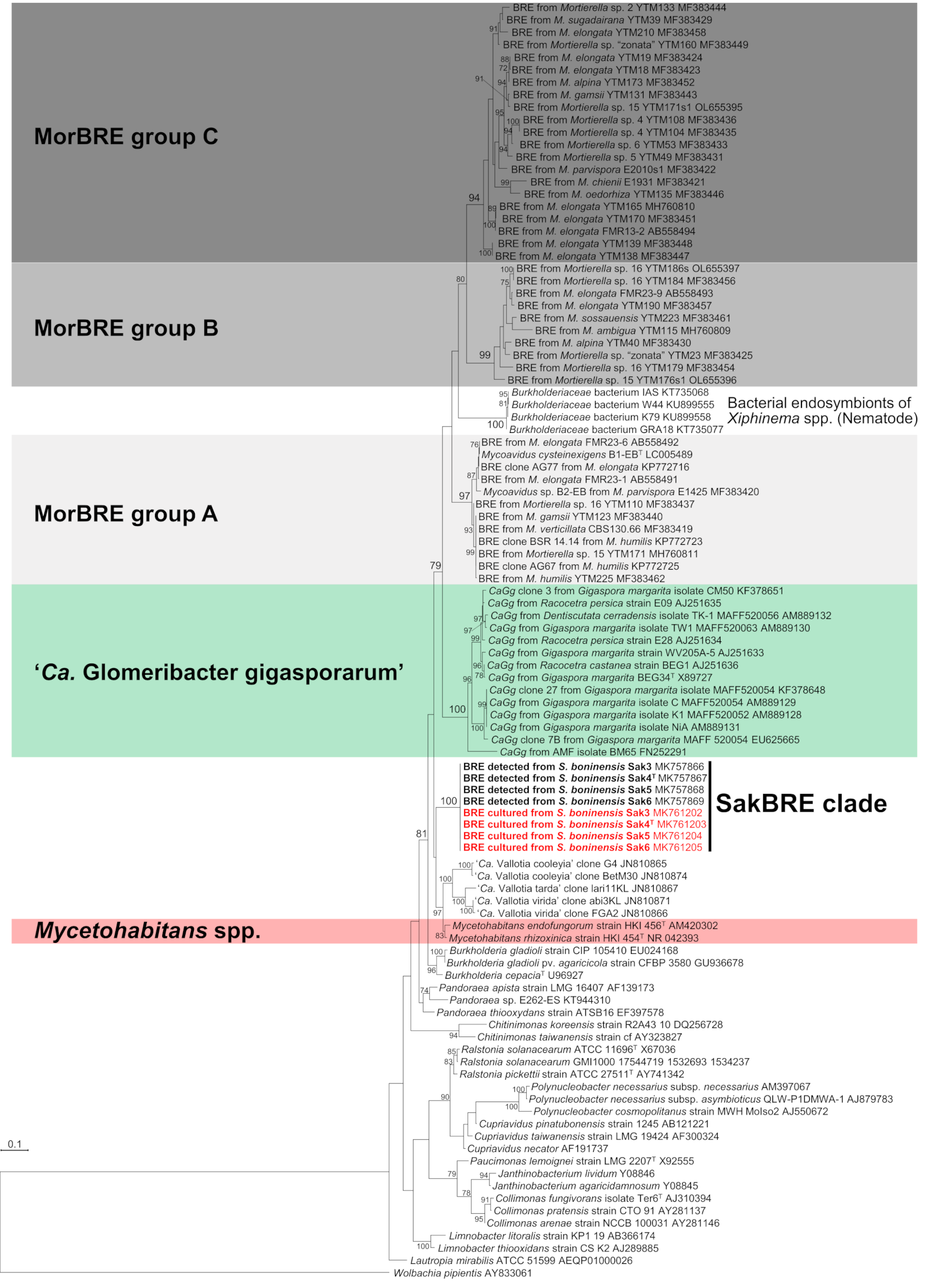

Supplement: Supplementary file 5 — Additional file 5 Fig. S4. The full version of Figure 6 shown in this study. [file 43008_2023_129_MOESM5_ESM.tiff]
